# Analysis of a novel X-ray lens for converging beam radiotherapy

**DOI:** 10.1038/s41598-021-98888-8

**Published:** 2021-09-28

**Authors:** Dirk A. Bartkoski, Aharon Bar-David, Michael Kleckner, Dragan Mirkovic, Ramesh Tailor, Jamshid Moradi-Kurdestany, Shirly Borukhin, Ze’ev Harel, Zeev Burshtein, Asaf Zuck, Mohammad Salehpour

**Affiliations:** 1 Department of Radiation Physics, Unit 1420, The University of Texas MD Anderson Cancer Center, 1400 Pressler St., Houston, TX USA; 2 Convergent R.N.R, 2 Yozma St., 3903202 Tirat Carmel, Israel; 3 Present Address: Israel Institute for Biological Research, 74100 Ness-Zionna, Israel

**Keywords:** Radiotherapy, Cancer therapy

## Abstract

We describe the development and analysis of a new teletherapy modality that, through a novel approach to targeted radiation delivery, has the potential to provide greater conformality than conventional photon-based treatments. The proposed system uses an X-ray lens to reflect photons from a conventional X-ray tube toward a focal spot. The resulting dose distributions have a highly localized peak dose, with lower doses in the converging radiation cone. Physical principles governing the design of this system are presented, along with a series of measurements analyzing various characteristics of the converging beam. The beam was designed to be nearly monoenergetic (~ 59 keV), with an energy bandwidth of approximately 10 keV allowing for treatment energies lower than conventional therapies. The focal spot was measured to be approximately 2.5 cm long and 4 mm wide. Mounting the proposed X-ray delivery system on a robotic arm would allow sub-millimeter accuracy in focal spot positioning, resulting in highly conformal dose distribution via the optimal placement of individual focal spots within the target volume. Aspects of this novel radiation beam are discussed considering their possible clinical application as a treatment approach that takes maximum advantage of the unique properties afforded by converging X-ray beam therapy.

## Introduction

Most conventional radiotherapy systems include radiation sources that produce diverging radiation beams that must then be collimated to achieve the correct target distribution. This divergence introduces a dose gradient that decreases with distance from the source. The work reported herein describes the potential of converging beam radiotherapy, which uses highly focused X-ray beams to produce unique dose distributions that facilitate placement of high doses within highly localized regions. The use of converging beams presents exciting new possibilities for achieving highly conformal treatments that better spare healthy tissues from inadvertent exposure to radiation. This report describes the fundamental principles of converging beams and a novel treatment device developed by Convergent Radiotherapy and RadioSurgery (CRnR; Tirat Carmel, Israel) that incorporates a Bragg-reflecting X-ray lens^1^. The basic design approach is described, as are measurements from a prototype system with clinically relevant materials, the experimental setup, and the clinical applicability of this novel system.

### Theory

The intensity of a simple radiation beam is affected by two primary factors: the attenuation of incident radiation as a result of interactions with particles in the medium and the geometric change of intensity due to the spread of radiation. For a monoenergetic beam of particles passing through a cross-sectional area *A(x)*, the charged particle equilibrium assumption^2^ allows a simple proportional relationship between particle fluence and dose deposited in a target at depth *x* relative to the surface at ${x}_{0}$ as

1 $$\frac{D\left(x\right)}{{D}_{0}}\propto \frac{A\left({x}_{0}\right)}{A\left(x\right)}{e}^{-\mu x}.$$

The linear attenuation coefficient $\mu$ describes the rate at which particles are lost due to beam-target interactions per unit distance^3^. Diverging beam geometry, where $A\left(x\right)>A({x}_{0})$, in conventional radiation treatment systems results in unavoidable beam loss and additional collimation for conformity. Conversely, a converging beam with a cross-sectional area that decreases in size during propagation such that $A\left(x\right)<A({x}_{0})$ results in a dose that increases with depth up to the focal point. Consequently, a converging or focused radiation beam reverses one of the primary mechanisms of dose loss.

**Figure 1:**
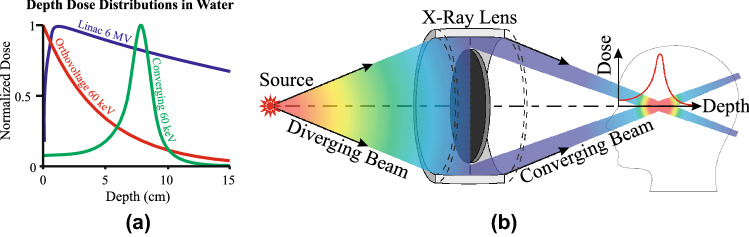
(**a**) Representative relative depth dose distributions for a diverging 6 MV photon beam, 60 keV orthovoltage beam, and 60 keV converging beam focused to a depth of 8 cm. (**b**) Conceptual illustration of the CRnR system, in which an X-ray lens converts a diverging beam into a converging beam, producing a localized dose peak within a target.

The relative dose distributions of a conventional diverging beam from a linear accelerator with a 60-keV orthovoltage beam, along with a converging quasi-monoenergetic 60-keV beam as produced by the CRnR system, are shown in Fig. 1. In Fig. 1a, the diverging beam shows the typical decrease of dose with depth after the initial build-up region (orthovoltage build-up is negligible), whereas the converging beam geometry, illustrated in Fig. 1b, exhibits an increase in dose up to the focal spot and then a decrease in dose as the beam becomes diverging.

A simple approximation for the attenuation in water, with an attenuation coefficient of $\mu  = \sim 0.206\;{\text{cm}}^{{ - 1}}$, would result in 50% attenuation of an initial 60-keV monoenergetic, parallel photon beam at a depth of approximately 3.4 cm, and relatively strong dose at shallow depths. For this reason, such low energies are not used in radiotherapy or radiosurgery. Converging radiation overcomes this problem, allowing the use of energies that are lower than conventional linear accelerators and the use of X-ray sources that are typically relegated to diagnostic imaging. Moreover, the dose peak resulting from the converging beam may be varied in depth, allowing the production of conformal 3D distributions through the summation of optimally placed focal spots. Converging beams have the potential to deliver far less localized dose before and after the target while delivering the maximum amount of dose at the target location.

The challenge in bringing converging X-rays to the clinic is to identify a method by which focused X-ray beams can be produced, because the refractive index for hard X-rays is below unity by 10^–6^, making the use of traditional focusing optics systems impractical^4^.

When high-energy photons enter a material, those that do not undergo inelastic processes (such as Compton scattering or the photoelectric effect) excite atomic electrons in such a way that the energy is re-irradiated with a nearly identical wavelength. Given a well-defined atomic structure with a separation of the atomic planes *d*, such as that found in crystalline solids, if the difference in path length between a scattered photon from adjacent planes is an integer multiple of the photon wavelength *λ*, then the scattered waves constructively interfere, resulting in a specularly reflected wave. This condition is known as Bragg’s law $n\lambda =2dsin{\theta }_{B}$ where *θ*_B_ is the Bragg reflection angle of the incident photon angle relative to the scattering plane*.*

Different interplanar distances can be obtained from the same crystal by selecting different sets of crystallographic planes formed by the atoms in the crystalline lattice resulting in different Bragg angles for the same photon energy. A crystal can be cut to present different crystallographic planes on its surface. For a unit cell with size *a,* the interplanar distance in a simple cubic crystal ${d}_{hkl}=a/\sqrt{{h}^{2}+{k}^{2}+{l}^{2}}$ can be expressed in terms of the Miller indices (*h, k, l*).

The theory of X-ray diffraction, in development since the early 1900s, is described extensively elsewhere^5–7^. Kinematic scattering theory, which assumes that the entire crystal coherently diffracts the incident electromagnetic wave without additional interference, results in a simple addition of diffracted waves from each unit cell with absorption from other scattering effects^6^. A dynamical approach, required ‘thick’ or strongly interacting crystals, introduces coupling between propagating waves, accounts for the influence of the crystal on the propagating beam.

**Figure 2:**
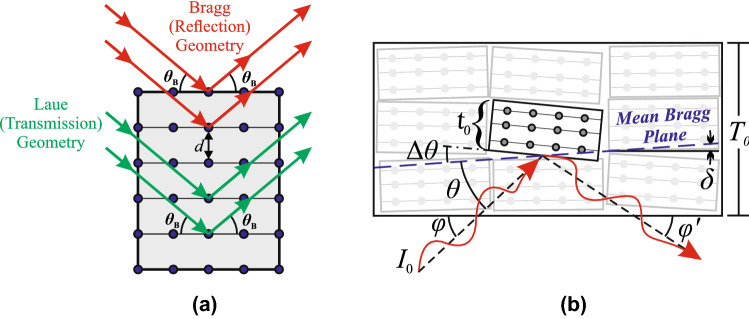
(**a**) Illustration of diffraction by a perfect crystal in Bragg reflection and Laue transmission geometries with interplanar distance d for Bragg angle $\uptheta _{{\text{B}}}$. (**b**) Depiction of a mosaic crystal with thickness T_0_ composed of individual crystallite blocks with thickness t_0_, each of which varies in angle $\Delta\uptheta$ around the mean Bragg reflecting plane, which has an angle $\updelta$ relative to the crystal surface. The incident beam, with an incident angle $\uptheta$ with respect to the mean Bragg plane, illustrates non-symmetric reflection geometry, in which the incident beam angle relative to crystal surface $\upvarphi$ and exit beam angle $\upvarphi ^{\prime }$ are not equal owing to the non-coplanar nature of the mean Bragg reflecting plane with the crystal surface.

The two possible diffraction geometries depicted in Fig. 2a are reflection (‘Bragg’) geometry, where incident and reflected X-rays pass through the same surface, and transmission (‘Laue’) geometry, where reflected X-rays exit the crystal through an opposing surface. Because the system described here involves reflection geometry, only solutions for reflection geometry are considered.

A perfect unbroken crystalline structure is known as a single-crystal. In general, metal crystals may be described as ‘mosaic single-crystals’ which are composed of many small, randomly oriented regions called crystallites, each of which displaying a perfect crystal lattice. The description of metallic crystals as being composed of highly aligned crystallites, illustrated in Fig. 2b, together with the dynamic analysis of Bragg diffraction, leads to the manifestation of the so called extinction effects.

Extinction refers to the reduction in intensity of the propagating wave resulting from various mechanisms, including interference effects from multiple internal reflections within a crystal structure as well as scattering effects due to consecutive crystallite layers^8^.

The extent of misorientation of crystallite blocks is known as the *mosaicity* and is characterized by a distribution of orientation angles $\Delta \theta$ of the crystallite blocks around the mean Bragg plane Fig. 2b. This distribution may have quite an irregular shape^9^ but is often assumed to follow a normal distribution where the full width at half maximum (FWHM) is the mosaicity. Extremely low mosaicities represent highly aligned crystallites and thus lead to almost perfect crystals. These crystals have larger extinction effects, as the dynamic theory predicts, but they also have lower angular and spectral acceptance for the incident beam, which results in reduced integrated diffracted power. On the other hand, an extremely high mosaicity approaches the limit of a polycrystalline material, where the Bragg reflection becomes extremely diffused.

Reflectivity is a function of the absorption of the material and the Bragg diffraction cross-section. Both kinematic and dynamic theories predict a range of reflections around the Bragg angle^10^. Because each small block within a mosaic crystal presents a slightly different Bragg angle, the cumulative effect of mosaicity is a larger range of reflected angles. Mosaicity also affects the reflected energy range, as can be seen by taking the derivative of the Bragg relation in the small angle approximation $2{d}_{hkl}{\theta }_{B}\approx hc/E$, leading to $\Delta {\theta }_{B}/{\theta }_{B}=\Delta E/E$. If the spread of Bragg angles $\Delta {\theta }_{B}$ is the mosaicity *m*, then

2 $$\Delta E=\frac{2{d}_{hkl}{E}^{2}m}{hc}.$$

The range of reflected energies increases proportionally with the mosaicity and with the square of the energy^11^.

Within the Bragg condition range, the probability of reflection approaches 1 for a perfect crystal and remains high even for mosaic crystals, such that the effective absorption is greatly reduced. The overall X-ray attenuation coefficient contains also the coherent part which includes the Bragg diffraction component. For single-crystals outside of the Bragg condition this component is very low, but at the Bragg condition it becomes very large. Thus, at the Bragg condition, most of the radiation is effectively diffracted while other absorption mechanisms become relatively less significant. The tiles were made thick enough to assure that the transmission of the Bragg component (at 60 keV) will be negligible relative to the incoming radiation. In practice the reflectivity varies with crystal quality; reflectivities for crystals used in this work are comparable with the expected values previously reported^12^ for 100 keV.

X-ray lenses made of perfect single crystals are rare because of the difficulty in manufacturing. The misorientation present in mosaic crystals translates into a higher angular acceptance for incoming radiation, which in turn leads to higher diffracted power and more efficient use of extended sources. Mosaic single crystals are therefore a more practical choice for the construction of X-ray lenses and are the types of crystals considered here.

### Design

Theoretically, a perfectly focused Bragg-diffracted X-ray beam can be produced with a point source using Rowland geometry^4^. Crystals with curved rather than flat diffracting planes have superior diffraction efficiency but are much more difficult and expensive to produce^12^ and are less practical for commercial application. The solution used in the CRnR system is to place small flat tiles coincident with the Rowland circle that are tilted in such a way to approximate the ideal Rowland geometry.

In three dimensions, the smooth, axially symmetric surface that provides perfect focusing of point source becomes an approximated surface that is segmented with flat tiles. Flat tiles are not inherently focusing. In fact, for a diverging source, the reflected beam is also diverging from each single tile, and only rays striking the tile surface located at the Rowland surface see the source with the required Bragg angle to be reflected to the intended focal spot. The result is less than perfect focusing. The tile geometry in the CRnR system has been optimized to mitigate this effect, as discussed below.

The X-ray source was a major factor driving the design of the CRnR system. Conventional external-beam photon therapies use the full spectrum of energy produced during the conversion of accelerated electron beams to X-rays. Because Bragg diffraction is valid for only a narrow range of energies, the X-ray source must be selected carefully to optimize the Bragg lens input energy. Except for a synchrotron light source, which would be impractical given the desired cost and size of the system, characteristic X-rays (the photons emitted when an outer shell electron loses energy to fill an inner shell vacancy after an ionization event) are a source of high-efficiency photons with well-defined energy.

For the CRnR prototype system, the Varex Imaging G-2090BI X-ray tube was chosen as the X-ray source, with modified electronics to adapt it for therapeutic use rather than imaging purposes. The Varex tube has a rotating anode with a 12° rhenium-tungsten target layer on graphite-backed molybdenum capable of 125-kV peak voltage with a maximum heat dissipation of 3700 W^13^. The G-2090BI photon energy spectrum resembles a typical tungsten X-ray tube spectrum, with a continuous bremsstrahlung component decreasing up to the maximum kVp and the characteristic X-ray peaks of tungsten^14^. To use the strongest emitted energy in a tungsten tube spectrum, the Kα_1_ transition with an energy of 59.32 keV was chosen as the energy around which to design the lens.

The choice of crystal material considered several factors, including a larger converging angle, greater penetration depth, better control of focal spot size and shape, and practicality for clinical application. Focal spot intensity and size are significantly influenced by the convergence angle (the Bragg angle in this case); an increase in the converging angle translates into increased focal spot intensity and smaller size. For implementation as a radiotherapy device, the system must have a reasonable radiation penetration depth considering the energy.

The cumulative effect of these criteria led to the following rationale for selecting the crystal material. The Bragg relation shows that the Bragg angle is inversely proportional to the X-ray energy and the crystal lattice interplanar distance; thus, larger convergence angles occur at smaller energies and smaller inter-planar distances. Material choice required optimizing the inter-planar distance characterized by the lattice constant, plane orientation, and mosaicity. An optimal mosaicity yields an energy bandwidth that is both wide enough to produce an adequate dose rate at the focal spot and narrow enough to not introduce a large spread.

Metal single crystal elements can meet these requirements of mosaicity owing to their inherent imperfections. In the work described here, selection of the material for the lens elements was based on experimental analysis of different single-crystal materials with preferable lattice plane orientations.

Crystal defects such as dislocations may be incorporated into the crystal during crystal growth, and in many cases, to an even larger extent during its processing into smaller segments and tiles. When present in excess, such defects reduce crystal reflectivity. However, at low concentration, dislocations can produce zoning by dividing the crystal volume into small perfect crystal blocks, thereby affecting the coherence properties of the diffracted beam traveling through the “ideally imperfect” mosaic crystal^15^.

For the first prototype, the material chosen was aluminum for all tiles used, with individual crystals chosen based on their mosaicity and cut parameters in accordance with the lens reflectivity requirements. Around 60 keV, there are several possible material choices with similar reflection properties^12^. Main considerations for choosing aluminum were heavily influenced by material availability, ease of single crystal growth, and other technical processing issues important for producing a commercially viable clinical product. Other materials are certainly an option for future investigation.

Further considerations in support of the choice of aluminum include that the intrinsic reflectivity via X-ray diffraction is approximately linear with the atomic number (fundamentally being directly proportional to the electrons density). However, the material X-ray absorption is approximately proportional to the fourth power of the atomic number^16^. Aluminum, being a low Z material, lets the radiation penetrate deeply into the crystal and, due to its mosaic structure, the penetrating X-rays have high probability of encountering crystallites with the correct Bragg orientation, thus, enhancing the overall diffraction.

The ensemble of mosaic crystallites that form the crystallographic planes are oriented up to a maximum angle $\Delta {\theta }_{max}$ around the average direction of the nominal Bragg plane. The mosaicity was measured for each tile with a rocking curve at 60 keV and is in the order of few tenths of a degree. As indicated by Eq. (2), energies around the ideal Bragg reflection energy may be reflected if they have an offset incident angle such that their wavelength still allows the Bragg diffraction equation to be satisfied. The energy bandwidth allows the reflection of additional energies, thereby increasing not only the reflected intensity from a polychromatic source but also the effective reflecting surface owing to the broadened allowed angular acceptance. Source size is also an important factor for reflected intensity.

An extended source, such as that from an x-ray tube, increases the angular distribution of incident photons on a given area of the tile surface. Photons with different incident angles within the same local area on the tile will encounter a range of mosaic crystallites such that photons emitted from different source locations may still be reflected towards the same point. Thus, within a certain limit, a longer tile may increase the focal spot intensity. In this work, the tile length, on the order of a few centimeters, was optimized based on the mosaicity such that the maximum crystallite tilt in the extremities of the tile still produced focused reflection angles for the wider range of photons from the extended source.

Although the mosaicity allows the reflected intensity from an extended tile to be increased, it also results in X-rays being reflected away from the focal point. The cumulative effect is that the distribution of X-rays around the focal point indicates the degree of mosaicity of the tile and size of the source. There are some direct beam effects which are shielded by blockers inside the lens aperture and outer shielding elements. Some photons diffracted in Laue geometry reflect as well but are primarily diffracted in different directions than the main Bragg reflections and subsequently collimated out by the shielding elements.

For flat tiles and a diverging source, the transverse width cannot contribute to focusing. Therefore, the optimal configuration is a ring of the largest possible number of transversely thin tiles. Manufacturing challenges limit the tile width to few mm, and the tile thickness is limited by the minimum thickness required for optimal diffraction. Practically, a thickness of > 1 mm is sufficient, and a thickness of a few millimeters was chosen for mechanical reasons. The optimization of tile size and mosaicity is non-trivial. Indeed, substantial effort in the development of the CRnR system has been devoted to growing and analyzing the crystal tiles for maximum output. A combination of simulation, experimental results, and practical considerations were used to arrive at the optimized tile dimensions which will be expounded upon in subsequent publications.

**Figure 3:**
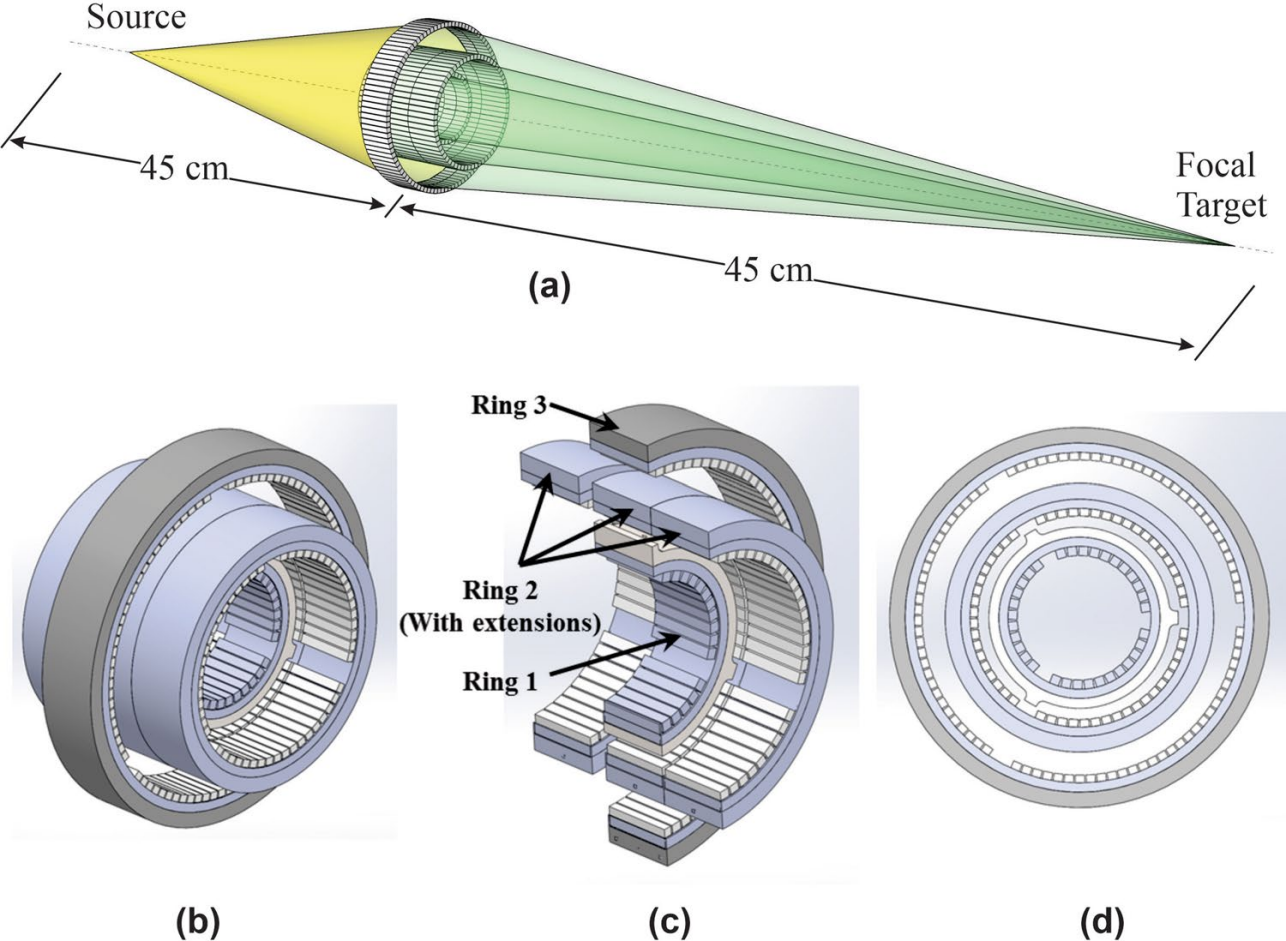
(**a**) CRnR lens concept. (**b**) Isometric view of the first-generation prototype CRnR X-ray lens. (**c**) Cut view with labels for each of the rings displaying the tile positions. (**d**) Beams-eye-view of the lens. Not shown is a lead block in the central aperture for preventing direct pass-through X-rays.

The optimized tile design and Rowland surface geometry led to design of the first-generation prototype lens (Fig. 3). The focal length was chosen such that the lens-to-target distance *F* would be 45 cm (Fig. 3a), which would allow adequate patient clearance throughout the motion of the system during treatment. The aperture width of the focusing cone of radiation at the exit of the shielding structure surrounding the lens is ~ 4.7 cm at a distance of 21 cm upstream of the focal spot.

The final design (Fig. 3b-d) consists of three concentric rings of Bragg-reflecting tiles aligned to the Rowland geometry, where each concentric ring consists of tiles with different crystallographic planes providing convergence of each ring to the same spot. The largest ring having a diameter of ~ 10 cm. Diffraction planes were chosen out of the first sets of allowed reflections (with highest reflectivity), with dependence on technical issues like crystal dimensions, ring spacing and geometrical shadowing. A complete lens is assembled with several concentric rings and some coaxial extensions with an average of approximately 60 tiles per ring with each ring being composed of identical tiles. The coaxial extensions of ring 2 serve to approximate the Rowland surface. Tiles were adjusted with a screw system that provide individual adjustment of each tile by orientation and radial position and then permanently fixed in place such that the lens becomes a single rigid unit. All the tiles reflections were directed to the focal spot with an accuracy of 130 um. An adjustment assembly also allows the lens to be aligned with the tube axis. A 4-mm-thick circular aperture block shields direct X-rays from the tube and permits the passage of only reflected X-rays (not shown).

## Methods

Measurement methods used to characterize the output intensity, dose distributions, and energy spectrum of the CRnR lens and the novel teletherapy beam are described in this section. Only relative and estimated deposited doses are presented. (Although absolute dose measurements have been obtained for several beam properties, an in-depth description of the method for calibrating the mono-energetic focused beam for this application is outside the scope of the current work and will be reported separately).

### Imager

One of the primary diagnostic tools used to analyze the CRnR beam was the PaxScan 2520DX Flat Panel Detector^17^. It has an amorphous silicon receptor with a CsI direct deposit conversion screen with an area of 19.5 × 24.4 cm, pixel pitch of 127 μm^2^, energy range of 40–160 kVp, and maximum frame rate of 12.5 fps. The flat panel detector allows real-time imaging of the beam, including the focal spot, which is a few millimeters in diameter. For the standard usage mode (1 × 1, 0.5 pF, VG1, 10 fps), the imager used in our measurements has a sensitivity of 4.91 counts/nGy which allows estimation of the dose delivered to air.

### Alignment

The highly localized nature of converging beam geometry mandated the use of an alignment system to allow accurate positioning of films and other imaging elements relative to the focal spot. This was accomplished by using three self-leveling line lasers (two opposing collinear lasers and one laser set perpendicular to the axis created by the opposing lasers) affixed to an external frame. The imager was used with a lead crosshair affixed to the imaging surface to image the focal spot transversely to the beam direction. The transverse position of the center of the focal spot was guaranteed by moving the imager until the X-ray image crosshairs were centered in the focal spot image. Longitudinal positioning of the focal spot was accomplished by moving the imager along the beam direction until the maximum number of counts was obtained. The external vertical and horizontal lasers, which were locked to the positions of the lead cross at the active layer of the imager, were subsequently used to position additional measurement tools.

### Film

Various forms of dosimetric film were used for qualitative and quantitative beam analysis. Gafchromic RTQA2 QA film (Ashland Inc., Bridgewater, NJ) was initially used for visualizing different planes of the CRnR beam. With a dynamic range of 0.02–8 Gy, high spatial resolution, and real-time self-developing properties, this film allows higher-resolution qualitative optical beam verification than that of the imager.

Most measurements were obtained with Gafchromic EBT3, which comprises a 28-μm active layer between 125-μm matte-surface polyester substrates. The film has a dynamic range of 0.1–20 Gy, minimal energy dependence ranging from 100 keV to > 1 MV, near-tissue equivalence, and a spatial resolution of 25 μm or less^18^. Films are read with an Epson 10000XL flat-bed scanner. All the film measurements were post-processed by using the pixel value pulled from the red channel of the RGB image scanned by the Epson 10000XL. Three separate scans of the same film were averaged to find the measured pixel value.

The film was scanned before irradiation to determine the pre-irradiation intensity *I*_*pre*_. Then, the film was scanned after irradiation to find the post-irradiation intensity *I*_*post*_. The net optical density^19^ was given by $OD = {\text{log}}\left( {\left( {I_{{pre}}  - I_{{opaq}} } \right)/\left( {I_{{post}}  - I_{{opaq}} } \right)} \right)$, where *I*_*opaq*_ is a calibration factor arising from the scanner. Dose was then determined from the film calibration curve. A 3rd order polynomial, forced to pass through origin, was fitted to the data with the equation

3 $$ Dose\left( {cGy} \right) = 897.6\left( {netOD} \right) - 123.5\left( {netOD} \right)^{2}  + 4639\left( {netOD} \right)^{3} . $$

**Figure 4:**
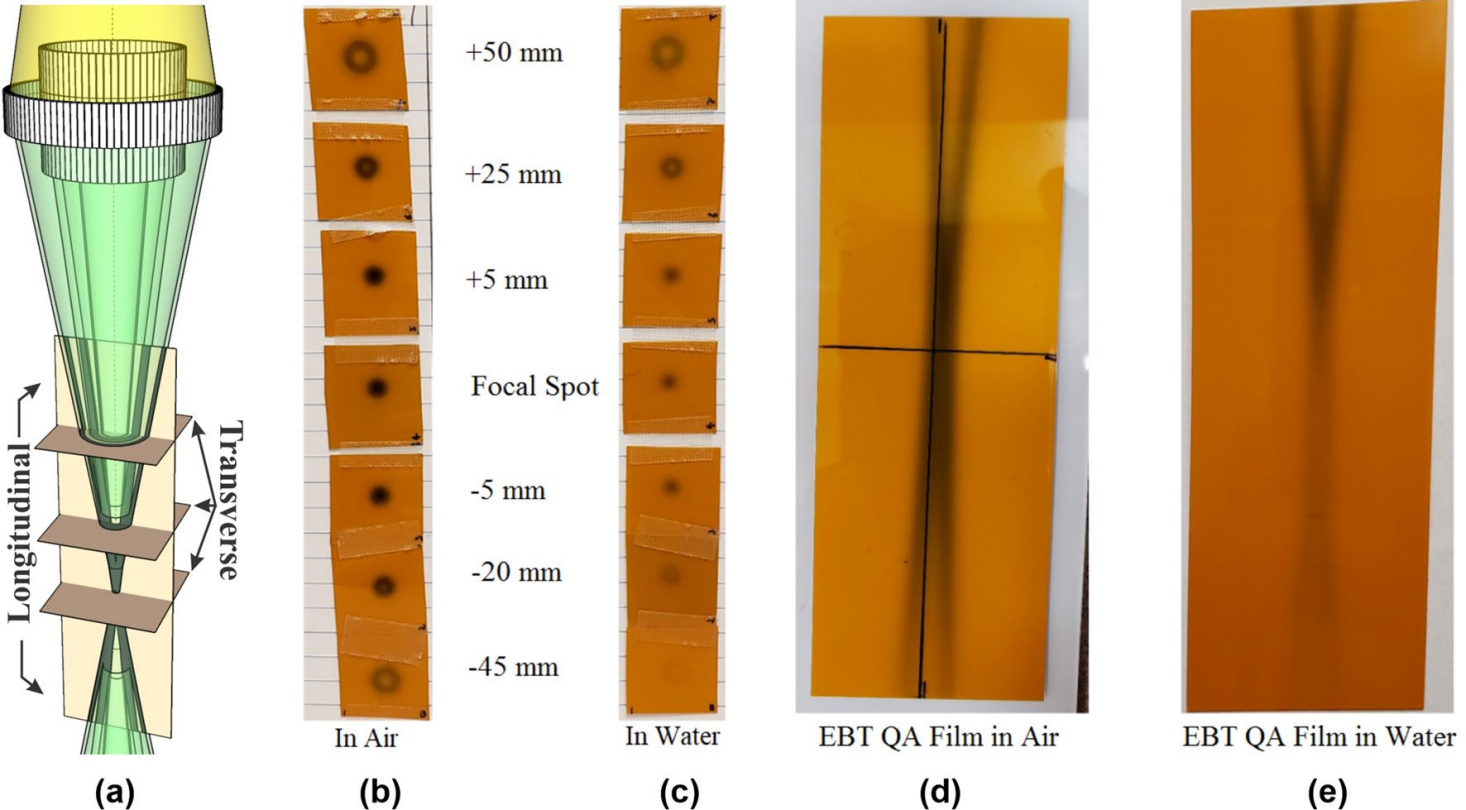
(**a**) Illustration of transverse and longitudinal film positions relative to the converging CRnR beam. (**b**) Transverse EBT-QA film measurements in air at positions above and below the focal spot. Positive numbers denote transverse positions upstream of the focal spot, whereas negative numbers denote transverse positions downstream of the focal spot. (**c**) Transverse EBT-QA film measurements in water. (**d**) Longitudinal EBT-QA film measurement in air. (**e**) Longitudinal EBT-QA film measurement in water.

Films were irradiated in two different orientations (see Fig. 4a). Transverse films, sometimes referred to as horizontal films, were oriented such that the film surface was perpendicular to the beam axis. Longitudinal, or vertical, films were oriented so that the surface was parallel to the beam axis and aligned so that the plane of the film passed through the center of the focal spot. Film measurements were obtained in air and in water; the in-water measurements were done with the films placed in a water-filled tank. Vertical films were placed such that the upstream edge of the film relative to the focal spot was coincident with the water surface. Transverse films were placed at various depths depending on the cross section of the beam to be visualized.

A conformal dose in a clinical treatment plan can be produced by scanning the focal spot over the desired target area while adjusting the beam intensity to overlap the focal spot gradients to generate uniform coverage over the desired region. The peaked nature of the focused beam allows the peak to be scanned longitudinally to produce a depth profile analogous to the spread-out Bragg peak produced by protons. This photon spread-out peak (PSOP) can be used to visualize the potential of the CRnR system to yield conformal dose distributions.

### Energy spectrum

Numerous spectrum measurements were obtained upstream and downstream of the lens by using an Amptek X-123 CdTe spectrometer. The X-123 is a cadmium-tellurium detector packaged with a digital pulse processor and multi-channel analyzer with a resolution of ~ 1.2 keV in the energy range of 5–150 keV^20^. Spectrometer measurements were corrected for loss in detection efficiency in CdTe detectors with increasing energy. Measurements also included escape peak correction, which compensates for photoelectric events in the detector just above the respective Cd and Te absorption edges that result in photons being emitted and escaping the detector, resulting in an excess of low-energy events^21^.

### Results

The novelty of the converging radiotherapy beam approach required a wide range of measurements to quantify and analyze the beam characteristics to facilitate implementing this approach into clinical environments. The measurements provided here do not include absolute dose results, which would require descriptions of beam calibration that are outside the scope of this report. The results shown here are intended to provide insight into the geometry and characteristics of the CRnR beam.

### Qualitative in-air measurements

Qualitative in-air measurements were taken with EBT-QA film to view the beam profiles. The results (shown in Fig. 4) are compared with EBT-QA film measurements in water to show the effect of attenuation. Subsequently, measurements taken with EBT3 film were used to determine the dose rate in air at the focal spot. For the maximum settings for the G-2090 tube at 125 kVp, 28.6 mA, and a 10-min delivery time, the imager sensitivity calibration revealed the dose rate to be 94 cGy/min.

### In-water measurements

#### Profile analysis

**Figure 5:**
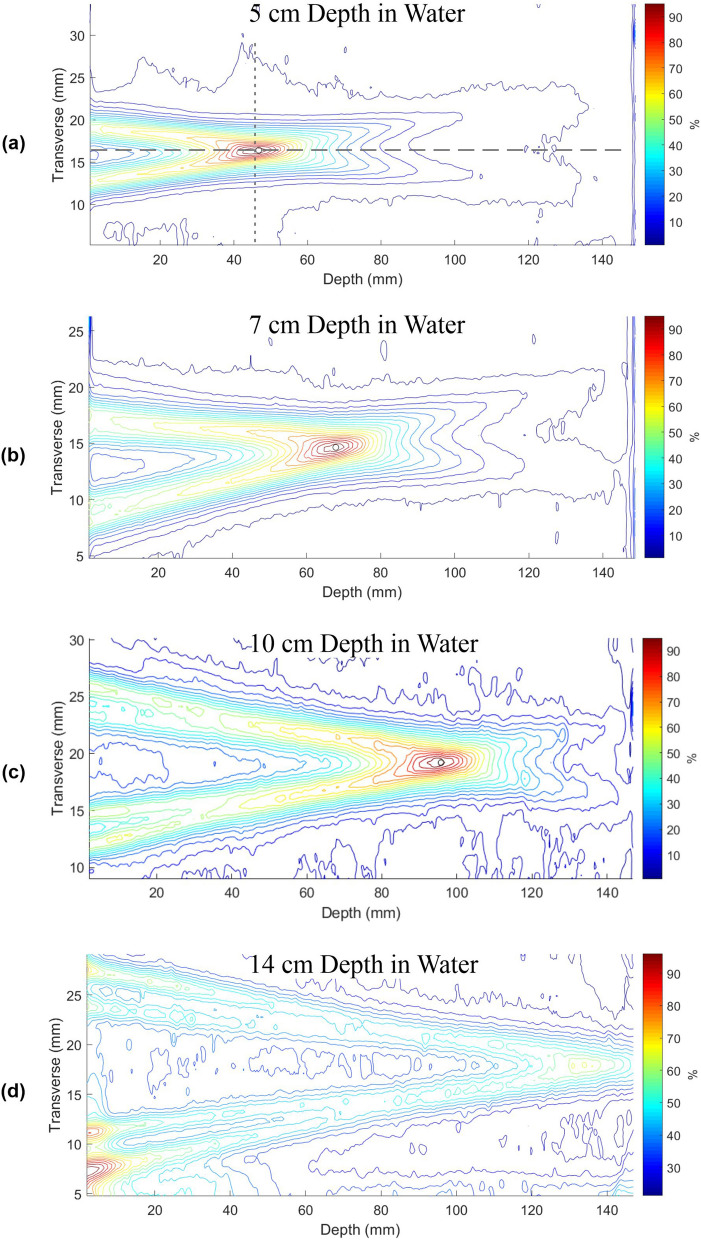
Relative isodose curves normalized to the peak dose for focused 60-kV X-rays. (**a**) Focal spot set to 5 cm depth in water. Long-dashed line represents the longitudinal profile line while the short-dashed line represents the transverse profile line. (**b**) Focal spot set to 7 cm depth in water. (**c**) Focal spot set to 10 cm depth in water. (**d**) Focal spot set to 14 cm depth in water.

Longitudinal distributions of the beam at various focal spot depths in water were taken. Profiles at four depths (5, 7, 10, and 14 cm) are displayed in Fig. 5. The focal spot dose relative to the ring profiles decreases with depth. For the greater depths, the individual beams from the separate lens rings can be resolved.

**Figure 6:**
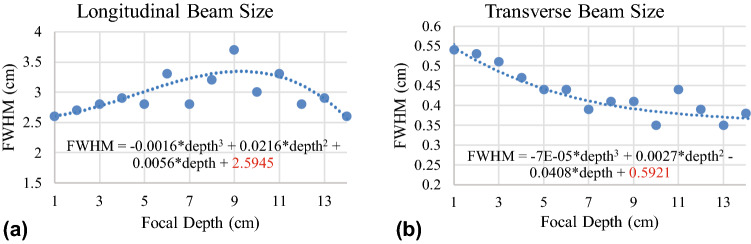
(**a**) Longitudinal focal spot beam size as defined by the central axis plot full width half maximum (FWHM). (**b**) Transverse focal spot beam size as defined by the FWHM perpendicular to the central axis at the focal spot maximum.

Longitudinal profiles of the beam at various focal spot depths in water were obtained using the vertical film measurements by selecting the dose as a function of depth along the central axis of converging cone passing through the focal spot. Transverse profiles were found by selecting a line of dose perpendicular to the beam passing through the focal spot. The primary contribution from the lens comes from the middle rings with the outer and inner rings having the additional effect of shortening the focal spot. As a result of scattering and relative attenuation of the ring contributions with depth, the longitudinal size of the focal spot increases with depth (Fig. 6a): the beam FWHM begins at 2.5 cm and increases to 3 cm at a depth of around 9 cm in water. The decrease in spot size above 9 cm is likely due to a combination of attenuation of lower energy photons with depth resulting in beam hardening and attenuation of non-optimally reflected photons. With depth, subsequently more non-optimally reflected photons are removed, leaving photons reflected from the optimal locations, i.e. the center of the tile. The cumulative result is that higher energy, ideally reflected photons are left at deeper depths resulting in a more ideally converging beam and smaller spot sizes. Transverse FWHM sizes for the focal spot are plotted in Fig. 6b, which shows a decrease in focal spot width, resulting in the FWHM decreasing from 6 to 4 mm. A 90% isodose region is an elongated spheroid approximately 1 mm wide and 8 mm long, resulting in a volume of 6.3 mm^3^.

The difference in the measured focal depth was also compared with the input depth or depth in air. For example, if the water-filled phantom was set such that the focal spot was to reach a depth of 5 cm, as it would in air, then the measured depth would be almost 4.5 cm. A relatively uniform 6-mm shift upstream of the focal spot was found that can be attributed to beam attenuation and scattering. For the shallowest depths, the focal spot increases to its stable value, which is intuitive considering the lack of beam attenuation and scattering at the target surface.

#### Photon spread-out peak

**Figure 7:**
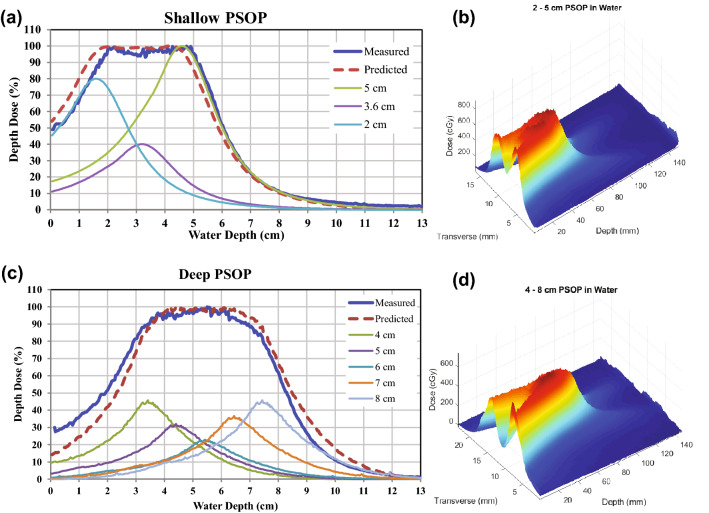
(**a**) Photon spread-out peaks (PSOPs) designed from 3 focal spots at depths of 2–5 cm in water. (**b**) Longitudinal surface dose map of the shallow PSOP. (**c**) PSOP designed from 5 focal spots at depths of 4–8 cm in water. (**d**) Longitudinal surface dose map of the deep PSOP.

A set of measurements was obtained to demonstrate dose “painting” with the focused X-ray s, to show the potential of the CRnR beam to produce a clinically relevant dose distribution. Two PSOPs were created (Fig. 7). The shallow PSOP (Fig. 7a,b) was created by summing the measured beam profiles at three depths (ranging from 2 to 5 cm) and scaling their relative strengths to achieve a uniform dose region. Experimentally, this was performed by adjusting the lens-to-target distance to change the focal spot depth and changing the X-ray source intensity to achieve the necessary relative total dose for each contributing profile. The predicted dose (dashed red line) was similar to the measured dose (solid blue line). The same procedure performed for a deeper PSOP (ranging from 4 to 8 cm) is shown in Fig. 7c. Unfortunately, the experimental setup used for these measurements had large positioning inaccuracies. However, even with this large experimental error, both the shallow and deep PSOPs showed good agreement between the expected and measured dose profiles. The experimental platform currently being assembled is expected to greatly improve these results.

### Focal spot spectrum

**Figure 8:**
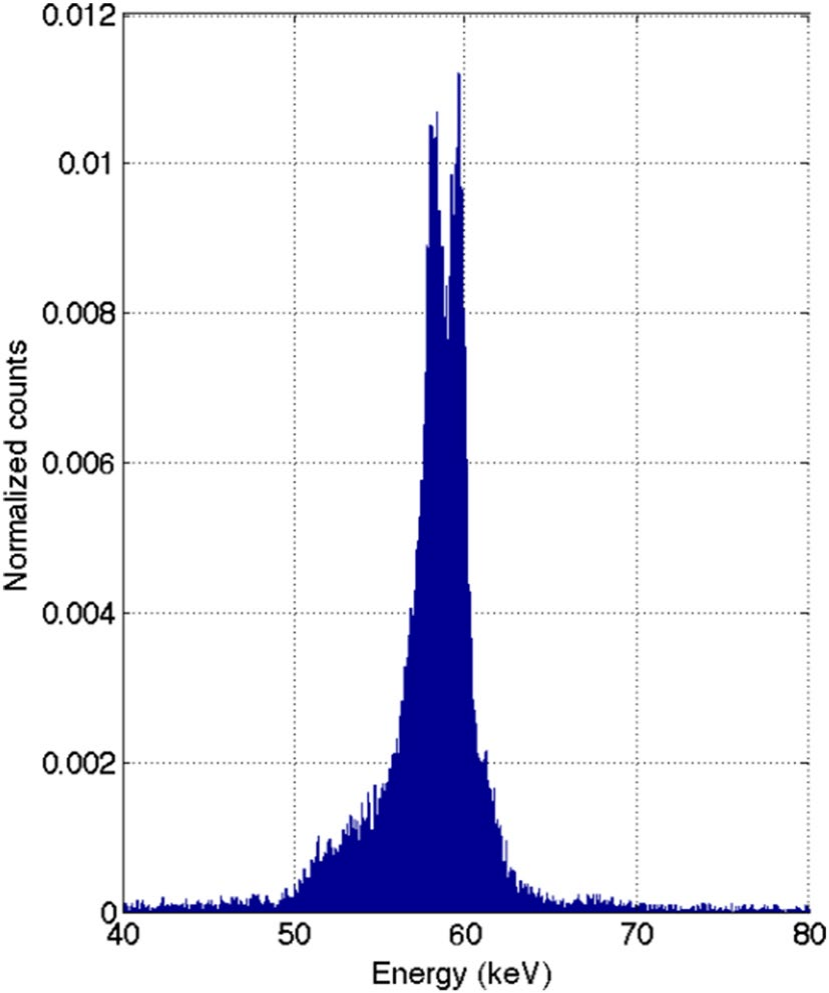
Energy spectrum of the Bragg-diffracted converging beam in air at the focal spot location.

The energy spectrum at the focal spot was measured to verify the monoenergetic nature of the CRnR beam (Fig. 8). Those measurements show peaks of 59.32 keV at Kα_1_ and 57.98 keV at Kα_2_, as expected. The spectrum exhibits a narrow band of energies, with the most significant contribution being between approximately 56 and 62 keV. This is a result of the various mechanisms contributing to reflection, including mosaicity. Although the CRnR beam is not purely monoenergetic, it has a sufficiently narrow energy bandwidth to be considered highly novel in comparison to conventional full-spectrum teletherapy modalities.

## Discussion

The CRnR X-ray lens and generator system was designed so that it can be mounted to a robotic arm, which allows the focal spot to be moved and orientated anywhere around the target. The target volume dose is then “painted” by irradiating a series of spots at the optimal positions and directions, such that the total dose distribution conformally covers the target and minimizes the dose to critical structures.

**Figure 9:**
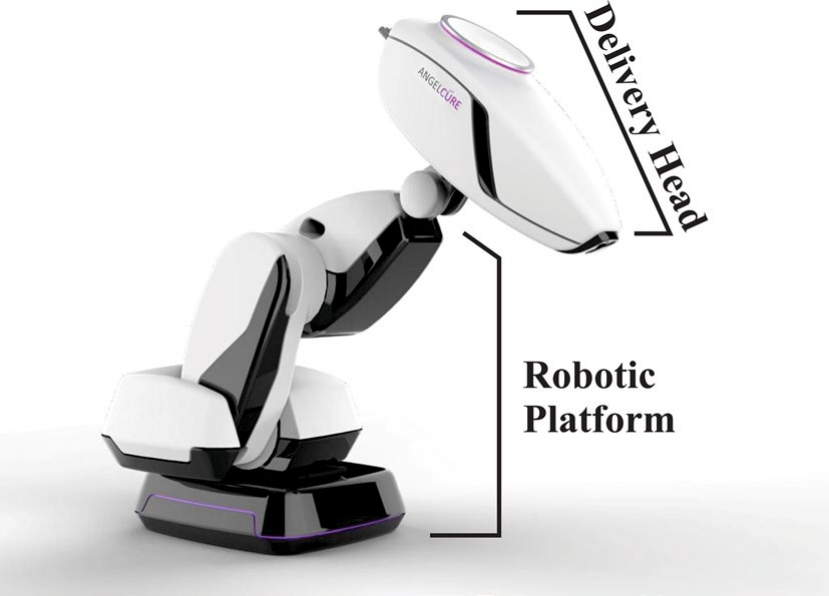
Early concept art showing the robotically mounted CRnR radiation platform with covers where the delivery head houses the X-ray and lens.

The CRnR system will be mounted on a KUKA KR QUANTEC 120 R1800 nano robot. With a maximum reach of 1803 mm and a payload of 120 kg, the R1800 is a 6-axis robotic arm capable of a positional accuracy of 60 µm and a minimum rotational axis speed of 86°/s^22^. An artist’s rendering of the planned clinical system, including the X-ray lens, generator, robotic platform, and casings, is shown in Fig. 9.

The CRnR system offers a unique approach to radiotherapy, which can be particularly challenging in pediatric patients because of the greater risk of developing secondary cancers due to radiation exposure^23^. Thus, the conformality and reduction of dose to healthy tissues are of prime importance in pediatric radiation oncology, and the CRnR converging beam is ideally suited for highly targeted delivery. This system may also be more widely applicable for children because they are physically smaller than adult patients.

The CRnR system would also be ideal for treating head and neck tumors in adult patients owing to the shallow depths of most of these targets. The converging nature of the CRnR beam would also be optimal for delivering radiation around highly sensitive structures such as the spinal column. The capability of converging beams to produce small spots could also be advantageous for ocular cancers, which are challenging to treating owing to the radiosensitivity of the optic nerve. This would also be an advantage for treating multiple small metastases, as many small spots may be treated quickly and efficiently with the robotic manipulation of the focal spot. Such high-precision dose delivery is also highly desirable in stereotactic radiosurgery, which uses high-intensity beams with very small margins of error. Other potential uses for the CRnR system include non-cancer–related therapies, such as treatments for age-related macular degeneration and denervation, which involve the use of highly localized radiation to treat nerves. Further, the conformality and mobility of the CRnR system could increase the availability of highly conformal treatment to a wider market. New applications for this novel approach are still being identified.

Efforts to improve the output of the CRnR system and increase the dose rate are under way. The scanned nature of the system’s dose distribution production would mean that increased dose rates would translate into improved delivery times and patient throughput.

A second-generation prototype lens is currently being developed with the goal of increasing the lens output by at least one order of magnitude. This goal will be accomplished by redesigning the lens structure, as extending the lens longitudinally is more advantageous than adding concentric rings. The first generation was comprising of concentric rings with increasing Miller indices whose reflectivity becomes lower as the index increases. In order to stay with the high reflectivity of low Miller indices, the design of the next generation will be with coaxial rings (rather than concentric) on the same Rowland circle. The crystal tile growth and quality assurance processes have also been improved, resulting in a higher diffraction efficiency per tile.

New type of tiles with a better collimated diffraction output are also under development. Incorporation of these tiles in the new lens design is expected to considerably reduce the dose rate at the target surface, which would improve the relative dose between the surface and focal spot by a factor of 3. The X-ray source being considered for the clinical implementation is the Varex MCS 72827, which increases the continuous output from 2 to 12 kW. This upgrade will help to improve the dose rate at the focal spot and the treatment delivery time for larger targets. Additional research to enhance both the Kα_1_ output from the X-ray tube and the non-Bragg energy range components of the input spectrum is also under way. Considering that non-Bragg energy range photons can contribute to scatter background contamination of the therapy beam, filtering out-of-band input photon energies will help to produce pristine focal spot distributions.

Also being developed is a sub-system for dose verification and beam interlocks that would provide a mechanism for real-time beam monitoring and beam shutoff capabilities in the event of software or hardware malfunctions. In conjunction with beam monitoring, onboard real-time imaging is being researched as a method of image-guided radiation therapy. One promising onboard imaging technique would involve using an unimpeded portion of the X-ray source passing through the center of the lens for imaging. This full-spectrum, diverging beam would be imaged by a companion detector array mounted on the robot downstream of the focal point, thereby allowing simultaneous imaging and treatment.

## Conclusion

This paper describes a treatment modality in which reflected X-rays are used to produce a converging photon monoenergetic photon beam, thereby producing highly localized dose distributions within a target volume with improved sparing of surrounding structures. Coupling such an X-ray delivery system with robotic manipulation allows conformal dose distributions to be “painted” in a manner not previously possible in conventional teletherapy modalities.

Measurements of the CRnR beam revealed a measured focal spot that is approximately 4 mm wide and 2.5 cm long. The beam was confirmed to be predominantly monoenergetic; its energy (about 59 keV) results in a useful depth of penetration in water of approximately 8 cm. A finalized system will offer unprecedented control over photon-based cancer treatment modalities. The CRnR converging beam approach represents a leap forward in this trend via the introduction of a new, possibly transformative approach to radiotherapy.

## References

[1] Burshtein, Z., Bar-David, A. & Harel, Z. System for X-ray irradiation of target volume (2013).

[2] Attix, F. H. *Introduction to Radiological Physics and Radiation Dosimetry*. (Wiley-VCH, 1987).

[3] Khan, F. M. *The physics of radiation therapy*. (Lippincott Williams & Wilkins, 2014).

[4] Schroer, C. & Lengeler, B. X-ray optics. In *Springer Handbook of Lasers and Optics* 1153–1164 (Springer, New York, 2007). 10.1007/978-0-387-30420-5_18.

[5] Authier A (2003). Dynamical Theory of X-ray Diffraction.

[6] Zachariasen WH (1945). The Theory of X-ray Diffraction in Crystals.

[7] Batterman BW, Cole H (1964). Dynamical diffraction of X rays by perfect crystals. Rev. Mod. Phys..

[8] Chandrasekhar S (1960). Extinction in X-ray crystallograpy. Adv. Phys..

[9] Schneider JR (1974). A γ-ray diffractometer: a tool for investigating mosaic structure. J. Appl. Crystallogr..

[10] Paternò G, Bellucci V, Camattari R, Guidi V (2015). Design study of a Laue lens for nuclear medicine. J. Appl. Crystallogr..

[11] Frontera F, von Ballmoos P (2010). Laue gamma-ray lenses for space astrophysics: Status and prospects. X-ray Opt. Instrum..

[12] Barrière N (2009). Experimental and theoretical study of the diffraction properties of various crystals for the realization of a soft gamma-ray Laue lens. J. Appl. Crystallogr..

[13] Varex Imaging. G-2090BI product datasheet. 1–18 (2017).

[14] Thompson, A. C. *et al. X-ray data booklet*. (CXRO, 2009).

[15] Bachmann KJ, Baldwin TO, Young FW (1970). Effect of dislocations on X-ray diffraction properties of copper. J. Appl. Phys..

[16] Evans, R. D. *The Atomic Nucleus*. (Krieger, 1982).

[17] Varex Imaging. PaxScan 2520DX Flat Panel Detector Datasheet. (2017).

[18] Ashland Inc. GAFCHROMIC DOSIMETRY MEDIA, TYPE EBT-3, specifications. 1–6 (2014).

[19] Devic S (2004). Dosimetric properties of improved GafChromic films for seven different digitizers. Med. Phys..

[20] AMPTEK Inc. X-123CdTe Complete X-ray & Gamma Ray Spectrometer. https://www.amptek.com/products/cdte-x-ray-and-gamma-ray-detectors/x-123-cdte-complete-x-ray-gamma-ray-spectrometer-with-cdte-detector#10 (2019).

[21] Redus, R. *CdTe Measurement of X-ray Tube Spectra: Escape Events* (Amptek Inc.). https://www.amptek.com/internal-products/cdte-measurement-of-x-ray-tube-spectra-escape-events (2008).

[22] KUKA AG. KUKA Robots for Low Payloads. 13–14 https://www.kuka.com/en-us/products/robotics-systems/industrial-robots/kr-quantec-nano (2019).

[23] Ng AK, Kenney LB, Gilbert ES, Travis LB (2010). Secondary malignancies across the age spectrum. Semin. Radiat. Oncol..

